# Supplementary material for: Identification of non-model mammal species using the MinION DNA sequencer from Oxford Nanopore
Source: PeerJ. 2024 Sep 25;12:e17887. doi: 10.7717/peerj.17887 (PMC11438440; doi:10.7717/peerj.17887)
Supplement: Supplemental Information 1 [file peerj-12-17887-s001.docx]

**Identification of non-model mammal species using the MinION DNA sequencer from Oxford Nanopore.**

Sara Velásquez-Restrepo^1^, Mariana Corrales-Orozco^1^, Nicolas D. Franco-Sierra^2,3^, Juan M. Martinez-Cerón^1^ & Juan F. Díaz-Nieto^1^

^1^Grupo de investigación Biodiversidad, Evolución y Conservación (BEC), Área de Recursos Naturales y Sostenibilidad, Escuela de Ciencias Aplicadas e Ingeniería, Universidad EAFIT, Medellín, Colombia.

^2^ Syndesis Health, FL 33408, United States of America.

^3^ VEDAS Corporación de Investigación e Innovación (VEDAS CII), Medellín 050024, Colombia.

Corresponding Author:

Juan F. Díaz-Nieto^1^

Carrera 49, Cl. 7 Sur #50, Medellín, Antioquia, Colombia.

Email address: [jdiazni@eafit.edu.co](mailto:jdiazni@eafit.edu.co)

**Apendix S1.** Listed below are all the localities where field work for this study was developed. Place names in italics indicate political department in Colombia. Geographical coordinates are given in parenthesis.

COLOMBIA

1. *Antioquia*, Hacienda La Sierra, 1350 m (5.9703453, -75.670357; Díaz-Nieto, 2021).
2. *Antioquia*, Pantanillo (Envigado), 2500 m (6.189161, -75.493359; Díaz-Nieto, 2021).
3. *Antioquia*, Parcelación la Miel (Caldas), 2600 m (6.108998, -75.592462; Díaz-Nieto, 2021).
4. *Antioquia*, Finca Socolao (Olaya), 2600 m (6.596916, -75.784348; Díaz-Nieto, 2022).
5. *Antioquia*, Universidad EAFIT (Medellín), 1400 m (6.200008, -75.579225; Díaz-Nieto, 2021)
